# Metabolic-suppressed cancer-associated fibroblasts limit the immune environment and survival in colorectal cancer with liver metastasis

**DOI:** 10.3389/fphar.2023.1212420

**Published:** 2023-08-31

**Authors:** Chenghao Wu, Shaobo Yu, Yanzhong Wang, Yuzhen Gao, Xinyou Xie, Jun Zhang

**Affiliations:** ^1^ Department of Clinical Laboratory, Sir Run Run Shaw Hospital of Zhejiang University School of Medicine, Hangzhou, Zhejiang, China; ^2^ Key Laboratory of Precision Medicine in Diagnosis and Monitoring Research of Zhejiang Province, Hangzhou, Zhejiang, China

**Keywords:** colorectal cancer liver metastases (CRLM), single cell transcriptome, cancer-associated fibroblasts, metabolism, immunity therapy

## Abstract

**Background:** Colorectal cancer liver metastasis is a major risk factor of poor outcomes, necessitating proactive interventions and treatments. Cancer-associated fibroblasts (CAFs) play essential roles in metastasis, with a focus on metabolic reprogramming. However, knowledge about associations between Cancer-associated fibroblasts metabolic phenotypes and immune cell is limited. This study uses single-cell and bulk transcriptomics data to decode roles of metabolism-related subtype of Cancer-associated fibroblasts and immune cells in liver metastasis, developing a CAF-related prognostic model for colorectal cancer liver metastases.

**Methods:** In this study, Cancer-associated fibroblasts metabolism-related phenotypes were screened using comprehensive datasets from The Cancer Genome Atlas and gene expression omnibus (GEO). Cox regression and Lasso regression were applied to identify prognostic genes related to Cancer-associated fibroblasts, and a model was constructed based on the Cancer-associated fibroblasts subtype gene score. Subsequently, functional, immunological, and clinical analyses were performed.

**Results:** The study demonstrated the metabotropic heterogeneity of Cancer-associated fibroblasts cells. Cancer-associated fibroblasts cells with varying metabolic states were found to exhibit significant differences in communications with different immune cells. Prognostic features based on Cancer-associated fibroblasts signature scores were found to be useful in determining the prognostic status of colorectal cancer patients with liver metastases. High immune activity and an enrichment of tumor-related pathways were observed in samples with high Cancer-associated fibroblasts signature scores. Furthermore, Cancer-associated fibroblasts signature score could be practical in guiding the selection of chemotherapeutic agents with higher sensitivity.

**Conclusion:** Our study identified a prognostic signature linked to metabotropic subtype of Cancer-associated fibroblasts. This signature has promising clinical implications in precision therapy for colorectal cancer liver metastases.

## Introduction

Liver is the most common site for metastasis of colorectal cancer (CRC), with approximately 50% of CRC patients developing liver metastases (LM) (Hu et al., 2021). Of these patients, 20%–30% have developed liver metastases at the time of initial diagnosis, and there is currently no effective treatment for colorectal cancer with liver metastases. One hallmark of cancer cells is metabolic reprogramming, which involves various metabolic changes that support faster proliferation. Glucose, nucleic acids, and lipids metabolism are all involved in this process (Faubert et al., 2020; Zhang et al., 2021).

The tumor microenvironment (TME) refers to the complex network of cellular and molecular components that surround and interact with tumor cells. It encompasses the intricate interplay between immune cells, such as T cells, B cells, natural killer cells, and macrophages, with tumor and stromal cells. Additionally, the extracellular matrix within the TME provides structural support and influences cellular behavior through dynamic crosstalk with the surrounding cells. The TME also contributes to the metastasis (Lee et al., 2020). Cancer-associated fibroblasts (CAFs) are crucial constituents in the microenvironment of solid tumors (Sahai et al., 2020). Compared to normal fibroblasts (NFs), CAFs exhibit lower contractility and higher ECM remodeling capacity, which secreting more pro-inflammatory mediators, matrix proteins, and immune regulators (Ishii et al., 2016; Lee et al., 2020; Sahai et al., 2020). Additionally, CAFs support tumorigenesis, progression, and metastasis in various ways through their interaction with cancer cells and immune cells.

Cellular metabolic reprogramming is a critical hallmark of malignancy and is most commonly observed in the tumor microenvironment, especially during metastasis. Metabolic reprogramming allows cancer cells to acquire cell-autonomous properties associated with enhanced invasiveness, which facilitate their escape (Faubert et al., 2020). Metabolic reprogramming has been reported to occur not only in tumor cells but also in the TME. Recent evidence has revealed the impact of metabolic interactions between CAFs and tumor cells on tumor metastasis (Faubert et al., 2020). The vast heterogeneity in the functions and sources of CAFs results in the existence of multiple subpopulations, each exhibiting partial functionality. Single-cell RNA sequencing (scRNA-seq), an emerging technology, enables the characterization of the intricate complexity and heterogeneity of distinct CAF subsets across various tumor types (Bartoschek et al., 2018; Friedman et al., 2020). Previous studies of the metastatic process have highlighted the concept of three major CAF subsets that can be dissected by their myofibroblast, inflammatory and/or immunomodulatory, and antigen-presenting activities (Banales et al., 2020; Xing et al., 2021). Nevertheless, the intricate interplay between CAFs and immune cells, as well as the effects of metabolic reprogramming during metastasis, are not yet fully understood, and further study is required to explore more specific CAF subtypes and their functions.

In this study, we identified distinct fibroblast subpopulations based on metabolic analysis at the single-cell level. We identified hub genes that are significantly linked to metabotropic subtypes of cancer associated fibroblasts. Finally, a CAF-related prognostic signature model was created using GEO datasets and demonstrated its roles in predicting outcomes and immunotherapy responses of patients with CRC and colorectal cancer liver metastasis.

## Methods

### Data source

Single-cell mRNA-sequencing data (Che et al., 2021) were collected from 6 CRC patients, patients numbered COL15, COL 17, and COL18 were patients who received chemotherapy and the others who were not. Bulk RNA-seq datasets GSE41258, GSE39582, GSE103479, GSE38832, GSE192667 and GSE15921 as well as TCGA bulk RNA-seq data with corresponding detailed clinical information were included in our analysis. This study adhered to the guidelines set by the TCGA and GEO databases.

### scRNA-seq data processing and analysis

scRNA-Seq data were processed by following Seurat pipelines in R (Hao et al., 2021). Briefly, genes expressed in less than 3 cells, as well as cells expressed less than 250 or more than 3000 genes were filtered out. Cells with high mitochondria and rRNA gene proportions were also excluded. Then, log-normalization was conducted to normalize the data from the 6 samples. The highly variable genes were identified using the FindVariableFeatures function, followed by scaling of all genes. PCA dimensionality reduction was performed to identify anchors. The cells were clustered with a resolution of 0.2. After initial sample integration, cell clustering and annotation, we generated a gene expression and phenotype matrix of 1897 CAF cells from all 111,292 cells.

### Metabolism score calculation and CAF subtyping

Metabolic activities of CAFs were evaluated by SCmetabolism packages (Wu et al., 2022). Each CAF cell was scored using the VISION algorithm, and finally the activity scores of the cell in different metabolic pathways were obtained. Second round clustering and subtyping of CAFs cells based on SCmetabolism scores were conducted to identify the heterogeneity of CAFs by Seurat pipelines.

### Cell communications analyses

Cell-cell communications based on ligand-receptor interactions were inferred by CellphoneDB (Efremova et al., 2020). To gain more critical cell-cell interactions in the colon cancer tumor microenvironment, we selected receptor-ligand pairs associated with hub genes for further analysis, aiming to explore potential interactions between immune cells. Significantly differential expressed ligand-receptor pairs (*p* < 0.05) were visualized.

### Trajectory analysis

Single-cell trajectories and determination of the continuous process of CAFs were analyzed by Monocle 2.0 package (v 2.10.0) (Jin et al., 2021). Pseudo-temporal analysis was applied to classify cells in pseudo-chronological order using the top 1000 differentially expressed genes in fibroblasts. Subsequently, a branch expression analysis model (BEAM analysis) was used to analyze branch fate-related genes.

### Survival analysis using CAFs-related features in bulk RNA-seq datasets

CAFs-associated gene signatures were generated by identifying the marker genes of all CAFs clusters. The activities of these genes in each sample of all CRC datasets were calculated using GSVA. Log-rank test and Cox proportional hazards regression were performed to explore the relationship between CAFs characteristics and patient prognosis, including overall survival (OS) and recurrence-free survival (RFS) rates. Cutoffs for different cell characteristics in different public datasets were determined by the survminer package and used for plotting Kaplan-Meier curves.

### Mutation analysis

Mutation data of CRC were obtained from the TCGA, and analyzed using the TCGAbiolinks package (Colaprico et al., 2016). Mutation landscape and lollipop plots were generated using maftools (Mayakonda et al., 2018).

### Construction and validation of CAF-related prognostic signatures

We predicted the prognostic characteristics of CRC patients by identifying CAF marker genes from scRNA-seq clusters. Using GSE192667 as training dataset, all CAF marker gene were investigated by univariate Cox regression models for the prognostic evaluation of OS time. Genes with significant prognostic effect (*p* < 0.05) were determined as candidate prognostic genes. The LASSO regression analysis was then used to identify the feature genes and optimize the model to prevent overfitting. Based on the coefficients generated from the LASSO analysis, a risk score was assigned to each colon cancer patient. Finally, we divided all colon cancer patients into high- and low-risk groups based on their risk score by the median. The association between the risk score and OS was assessed using Kaplan-Meier analysis. Heatmaps were generated to visualize the associations between CAF risk scores and candidate genes. The time-dependent prediction accuracy of our model in the training, internal, and external test datasets was evaluated using AUC.

### Functional analyses of CAF subtypes

After obtaining differentially expressed genes between CAF subtypes, Metascape (https://Metascape.org) was used for gene set enrichment analysis. To estimate the infiltrating immune cells in the tumor microenvironment, CIBERSORT package was used to infer the relative abundance of immune cells in each sample (Yoshihara et al., 2013). Gene sets of tumor-associated canonical pathways were obtained from previous study (Sanchez-Vega et al., 2018). Activities of these gene sets were generated by single cell gene set enrichment analysis (ssGSEA) for cell state assessment of each tumor sample.

### Immunohistochemistry (IHC) staining

Tumor and adjacent tissue samples were fixed in formalin and embedded in paraffin. For IHC staining, sliced samples were deparaffinized and rehydrated. After that, antigen retrieval was performed, and normal goat serum was used for 10 min at room temperature to block non-specific binding site. Each slide was treated with mouse monoclonal anti-human NNMT antibody (1E7, diluted 1:1400) and incubated in 37°C for 40 min. Then, slides were incubated with biotinylated goat anti-mouse antibody for 30 min, and chromogenic reaction was carried out using a diaminobenzidine (DAB) Substrate Kit. Finally, Digital slide scanning system (KF-PRO-005) was used to capture images of IHC. The staining scores of NNMT protein expression were evaluated by two independent pathologists based on their clinical information. The protein expression levels were classified as 0 (no staining), 1+ (weak staining), 2+ (moderate staining), or 3+ (intense staining), and the staining score was calculated by integrating the percentage of positive cells and the respective intensity scores. The staining score ranged from a minimum value of 0 to a maximum value of 300.

### Chemotherapy sensitivity and immunotherapy response prediction

Chemosensitivities of high and low CAF score groups were evaluated by oncoPredict (Maeser et al., 2021). Briefly, a ridge regression model with 10-fold cross-validation was built to infer half-maximal inhibitory concentrations (IC_50_) Value. Pharmacogenomics database Genomics of Cancer Drug Sensitivity (GDSC; https://www.cancerrxgene.org) (Yang et al., 2013) was used to assess the response of CRC patients to chemotherapy. In addition, the Tumor Immune Dysfunction and Exclusion (TIDE) algorithm (Jiang et al., 2018) was implemented to predict immune checkpoint blockade treatment response between the two groups.

Statistical analyses were conducted using R software (v 4.1.0) and data visualizations were generated using appropriate R packages. Non-parametric tests such as the Wilcoxon rank-sum test were used for comparing two groups of continuous variables that did not follow normal distributions, while the Kruskal–Wallis test was used for testing three or more groups. Cox regression was employed to calculate hazard ratios (HR), and Kaplan-Meier analysis was used for prognostic evaluation. Statistical significance was set at a two-sided *p*-value of <0.05. Spearman’s correlations were determined for correlation analysis (*p* < 0.05, **p < 0.01).

## Results

### Metabolic subtypes of fibroblast in CRC using scRNA-seq data

**FIGURE 1:**
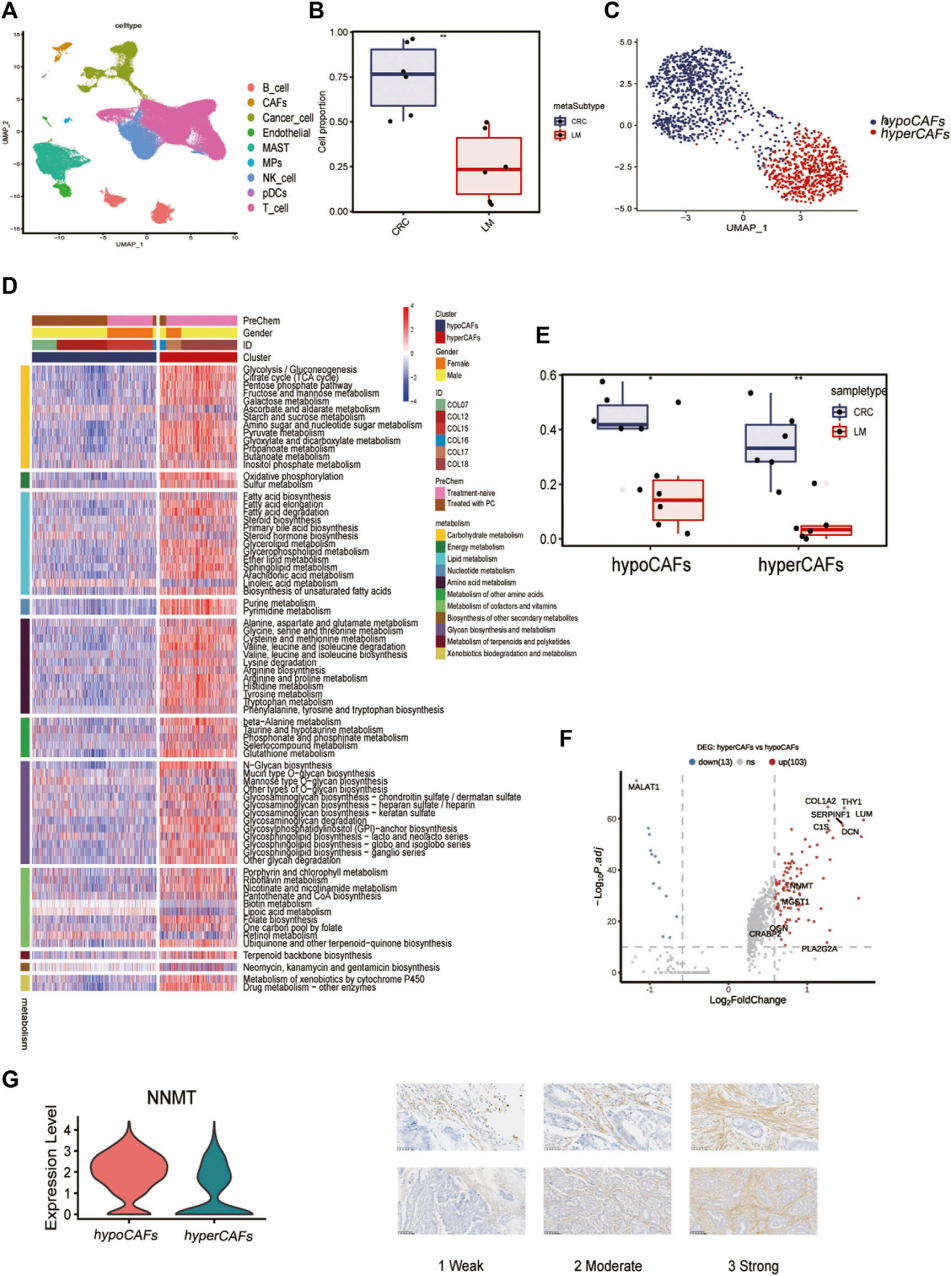
Single-cell transcriptome analysis and functions of CAF-related genes. **(A)** Nine cell subsets are shown. **(B)** Boxplot showing the difference between primary and metastatic lesions. **(C)** UMAP plot showing the metabolic grouping of CAF cells. **(D)** Heat map showing differences in 11 metabolic pathways between different metabolic groups. **(E)** Boxplots showing the differences between primary CRC and liver metastases in hypoCAFs and hyperCAFs. **(F)** Volcano plot highlighting the signature genes of different clusters. **(G)** Immunohistochemistry showing NNMT gene expression in fibroblasts.

Six samples obtained from the SMC cohort (GSE178318) were included in our study, which underwent quality control based on cell characteristics and mitochondrial and ribosomal gene expression. Subsequently, dimensionality reduction was performed to classify all cells. T-Distributed Random Neighborhood Embedding (t-SNE) was used to divide the cells into 9 major clusters and 32 more detailed minor clusters (Figure 1A). To further explore the metabolic signature of tumor fibroblasts, we calculated their metabolic signature pathways scores using SCmetabolism and clustered these CAFs based on these scores. Two fibroblast subclusters were identified (Figure 1C), and the important differentially expressed pathways for each cluster were visualized by heatmap for 11 metabolic pathways, including carbohydrate metabolism, energy metabolism, lipid metabolism and other nutrients metabolism (Figure 1D). According to the expression activities of metabolic pathways, two fibroblast subclusters were determined as hypermetabolism CAFs (hyperCAFs) and hypometabolism CAFs (hypoCAFs).

In primary CRC, total CAFs were significantly more abundant than in liver metastases (Figure 1B). Proportions of both CAFs subtypes also were greater in primary tumors than in metastases (Figure 1E). CAF in CRC patients showed significant heterogeneity in different sample, COL12 having a more even proportion in CAFs cells than others (Supplementary Figure S1). However, the proportion of hypoCAFs in other patients was significantly lower than hyperCAFs.

We identified differential expressed genes between clusters (Figure 1F). Hypometabolic CAFs shows higher expression of genes involved in myogenesis and pericyte-associated markers, such as MALAT1. While hypermetabolic CAFs highly expressed THY1, COL1A2, and some metabolism-related genes, such as PLA2G2A and NNMT. We focused on nicotinamide N-methyltransferase (NNMT), which is a cytosolic enzyme that has been identified as a significant metabolic regulator of cancer-associated fibroblasts (Eckert et al., 2019). Our findings were supported by immunohistochemical staining, which revealed that NNMT is predominantly expressed in fibroblasts (Figure 1G). The expression of other metabolism-related genes, including CRABP2, PLA2G2A, OGN, were all highly expressed in hyperCAFs subtypes (Supplementary Figure S2).

### Functional heterogeneity, trajectory, cell-cell communication and transcription factors analysis of CAFs in CRC

**FIGURE 2:**
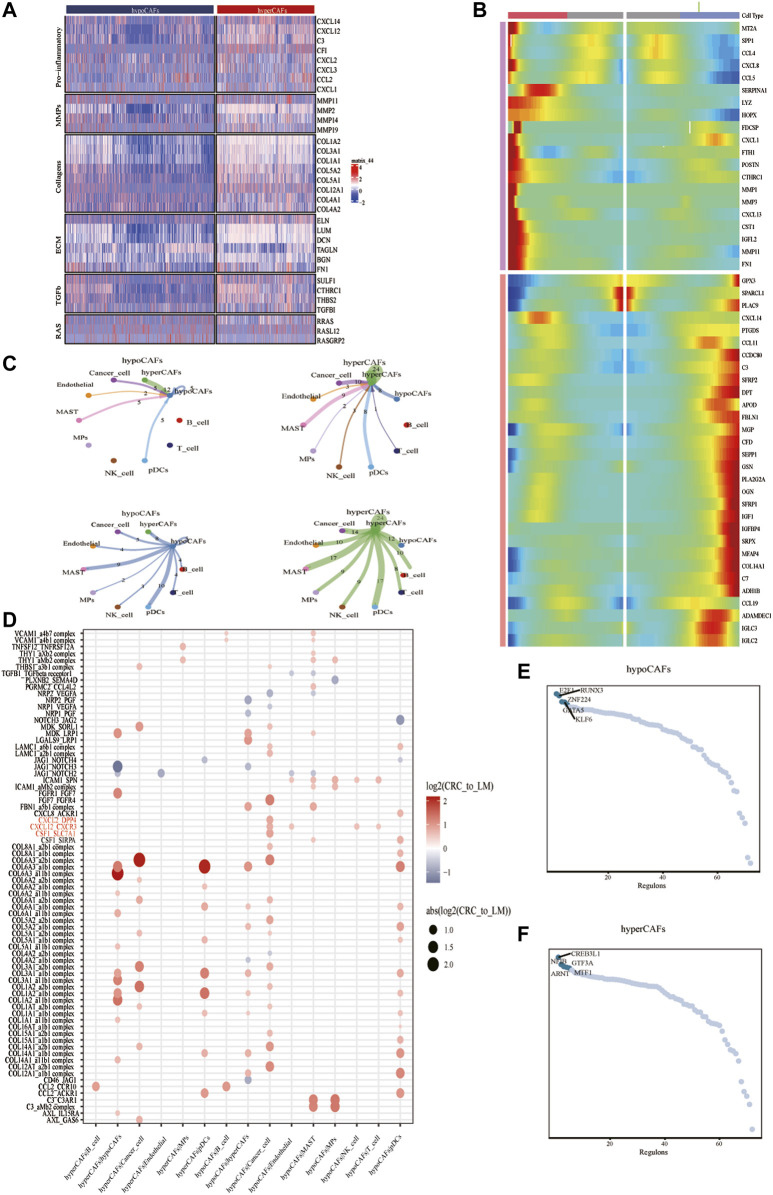
Analysis of cellular communication in the TME of CAF cell populations in other different metabolic states. **(A)** Correlations between CAF clustering characteristics and cytokines including chemokines, interleukins, and other cytokines. **(B)** Heat map showing that CAF cells can exhibit two expression patterns after differentiation. **(C)** Shows the number of ligand-receptor pairs between CAF and other subclusters. **(D)** Shows a comparison of specific ligand-receptors between CAF clusters and other subclusters in primary and liver metastases. **(E)** Regulator-specific scores for regulon activity of CAF isoforms.

To describe and explain the functional heterogeneity of the two CAF subpopulations, several sets of genes characterizing the related functions of CAFs were used. Heatmap showed that different metabolic CAF subpopulations were characterized by significant differences in the expression of collagen genes, angiogenesis genes, smooth muscle-related contractile genes, and members of the RAS superfamily (Figure 2A).

Trajectory analysis of CAFs was performed based on the Monocle 2 algorithm to infer the maturation process of CAFs (Figure 2B, Supplementary Figure S3). In particular, we dissected gene patterns involved in CRC cell state transitions. Cell-to-cell communication analysis revealed large-scale interactions between the two CAF subpopulations and other cell types. Hypometabolism CAFs had cellular interactions among hypometabolism CAFs, tumor cells, and endothelial cells (Figure 2C), While hypermetabolism CAFs had the strongest interaction on tumor cells and mast cells (Figure 2C).

We compared different CAF subtypes between CRC and metastasis with genes related to tumor proliferation, metastasis, and progression pathways, to explore whether significant interactions were observed among different cell subsets. The results showed that CAFs, tumor cells, and B cells can participate in a series of functional interactions involving CXCL12 receptor-mediated APP, COPA, and MIF signaling (Figure 2D).

Activity of each transcription factor (TF) and its regulated genes were also inferred in both CAF subtypes. By comparing regulator specificity scores (RSS), we examined key regulators for each cell type and visualized the top 5 regulators (Figures 2E,F). E2F1, RUNX3, and ZNF224 were identified as the top regulators for hypometabolism CAFs, while CREB3L1, NFIB, ARNT, and GTF3A were identified as the top regulators for hypermetabolism CAFs.

### Functional and prognostic role of CAF metabolic subtypes signature genes in metastatic CRC

**FIGURE 3:**
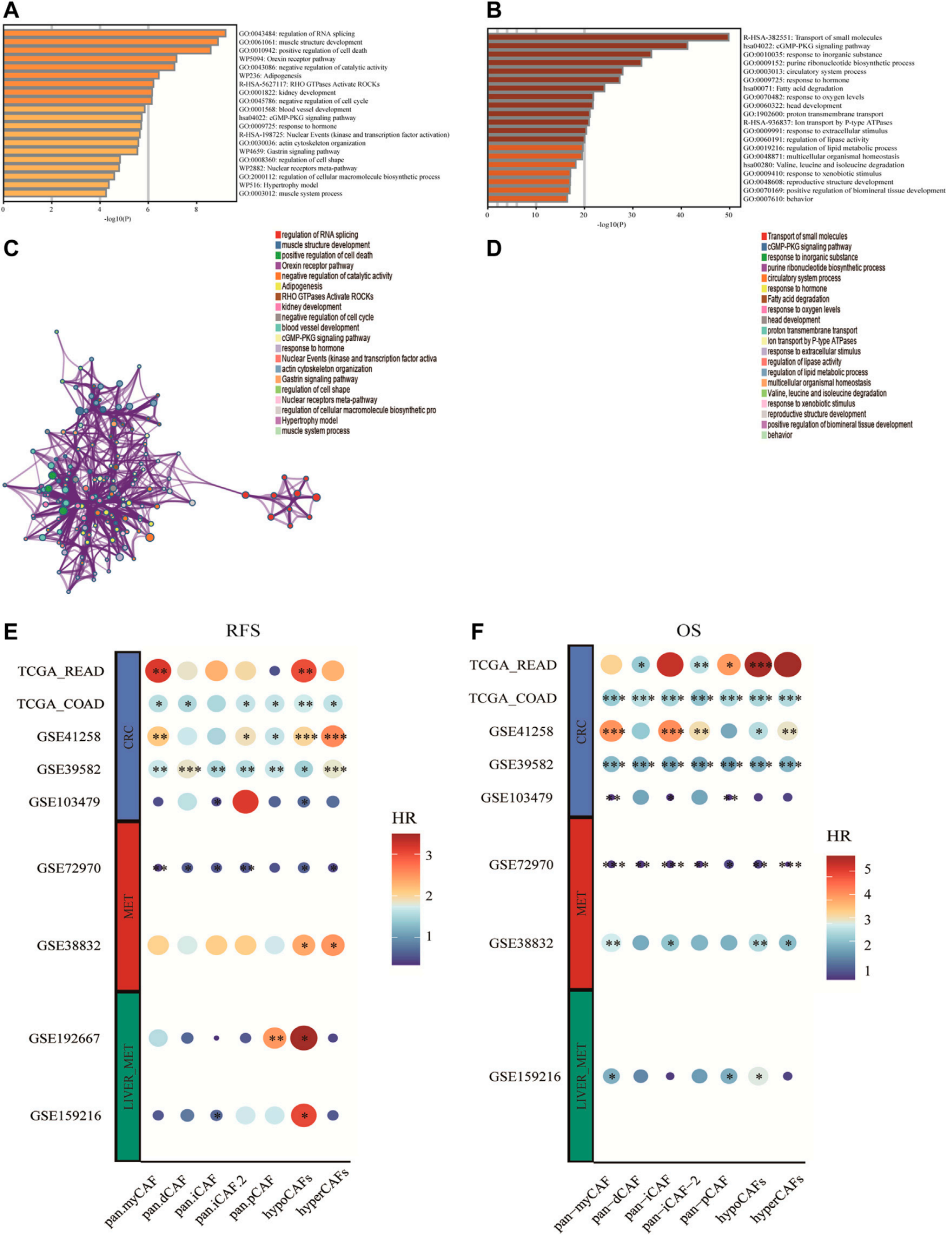
Enrichment analysis of CAF cell subgroups and prognosis **(A,B)** Enrichment analysis and corresponding networks of differentially expressed associated genes in CAF subgroups. **(C,D)** Prognosis of CAF cluster (GSVA score). The cut-offs were calculated by the survival R packages. RFS analysis (data from 9 CRC cohorts); B OS analysis (data from 8 CRC cohorts).

Based on differentially expressed genes (DEG) (Supplementary Table S1) between two CAF subtypes, we performed the functional enrichment analysis using the online gene ontology (GO) enrichment analysis tool Matascape. Highly expressed genes of hypometabolism CAFs were enriched in the regulation of RNA splicing and muscle structure development GO terms, while genes of hypermetabolism CAFs were enriched in the transport of small molecules, cGMP-PKG signaling pathway, and some metabolism-related pathways such as fatty acid degradation (Figures 3A–D).

To investigate the association between CAF subtypes signatures and overall survival (OS) and recurrence-free survival (RFS) of CRC patients, we computed metabolic subtype scores for CAF subtypes by GSVA. Prognostic analysis was performed on all differentially expressed genes (DEGs) across nine publicly available cohorts that were classified into three types according to the metastasis status (i.e., primary tumor cohort, tumor cohort with metastases, and tumor cohort with liver metastases). We conducted a meta-analysis to obtain stable prognostic results for CAF subtypes, and compared our metabolic subtypes with Pan-CAF signatures derived from previous studies (Galbo et al., 2021). Our analysis revealed significant differences between subgroups in hypometabolism CAF scores compared to hypermetabolism CAF scores, in relation to RFS and OS. As a result, we defined hypometabolism CAF-type cells as a class of cells that are specific to colorectal cancer patients with LM.

### Construction and validation of metabolism-CAF score based on metabolism subtypes of CAFs

**FIGURE 4:**
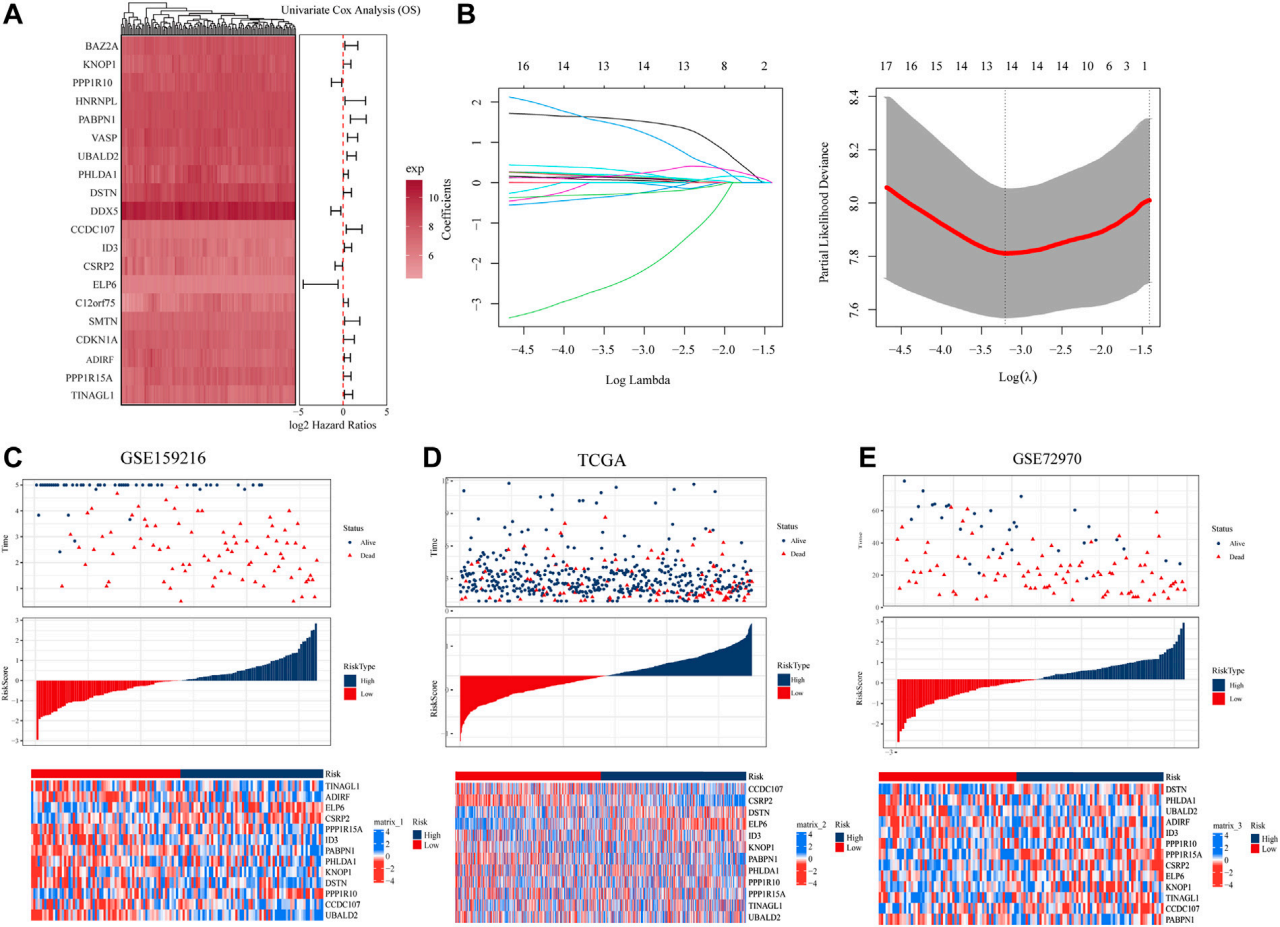
Construction and evaluation of prognostic risk model. **(A)** In the GSE159216 dataset, 20 genes were selected for analysis by univariate Cox regression. **(B)** The ten-fold cross-validation and LASSO coefficient distribution used to screen the optimal parameter (lambda) was determined by the optimal lambda. **(C)** Differences in overall survival between high-risk and low-risk groups in the GSE159216 training cohort. **(D)** Difference in overall survival between high-risk and low-risk groups in the TCGA validation cohort. **(E)** Differences in overall survival between high-risk and low-risk groups in the GSE72970 validation cohort.

To explore the prognostic genes associated with hypoCAF, we selected 20 genes based on the univariate Cox regression analysis in the GSE159216 dataset (Figure 4A). These 20 genes underwent Lasso-Cox regression analysis with 10-fold cross-validation to generate the optimal model, which highlighted 13 genes with the smallest partial likelihood deviation and optimal regression efficiency, including TINAGL1, ADIRF, ELP6, CSRP2, PPP1R15A, PABPN1, PHLDA1, ID3, KNOP1, DSTN, PPP1R10, CCDC107, and UBALD2. The risk score was calculated using the formula, and applied to GSE159216, TCGA, and GSE72970 datasets. Results indicated that colorectal cancer patients with high risk score had a higher mortality rate (Figures 4C–E). Heatmap results showed significant differences in the 13 genes expression between two risk score groups.

**FIGURE 5:**
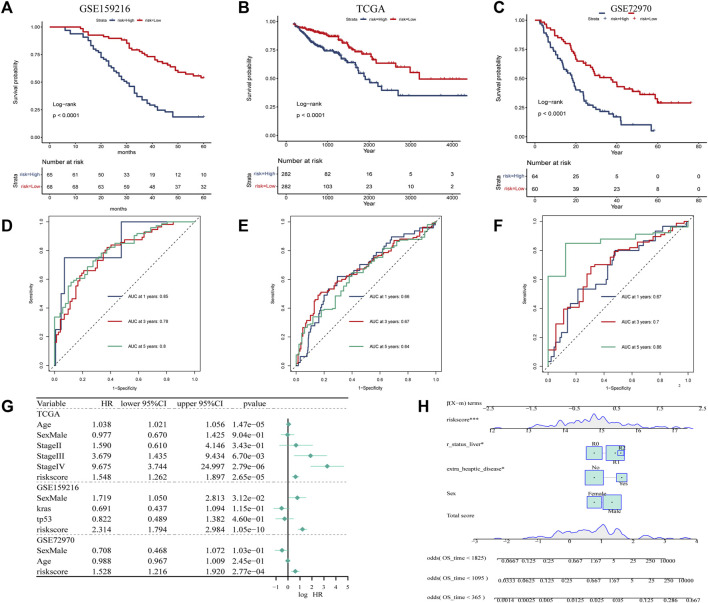
Creation of CAF-related prognosis and nomogram. **(A–C)** Kaplan-Meier prognostic analysis of signatures across training, testing, and entire datasets. **(D–F)** Time-dependent ROC signature curves in the training, testing GSE159216, TCGA and GSE72970 datasets. **(G)** Univariate Cox regression in the GSE159216, TCGA and GSE72970 cohorts.

According to KM curves, patients in the high risk score group had lower survival rates than those in the low risk score group (Figures 5A–C). We also performed time-dependent ROC analysis, with the AUC values of our model in the training set for 1-year, 3-year, and 5-year overall survival being 0.85, 0.78, and 0.80, respectively (Figure Figure5A). In TCGA, the AUC for our 1-, 3-, and 5-year survival models were 0.66, 0.67, and 0.64, respectively, while in the GSE72970 dataset, the AUC values for 1-, 3-, and 5-year survival models were 0.74, 0.75, and 0.74, respectively. Cox regression analysis in three cohorts showed CAF score model would be an independent prognostic marker for CRC with LM (Figure 5G). We developed a nomogram based on CAF score to predict overall survival in CRC patients at 1, 3, and 5 years (Figure 5H). The calibration curves for each time point showed excellent predictive performance.

### Biological features of Metabolism-CAF score

**FIGURE 6:**
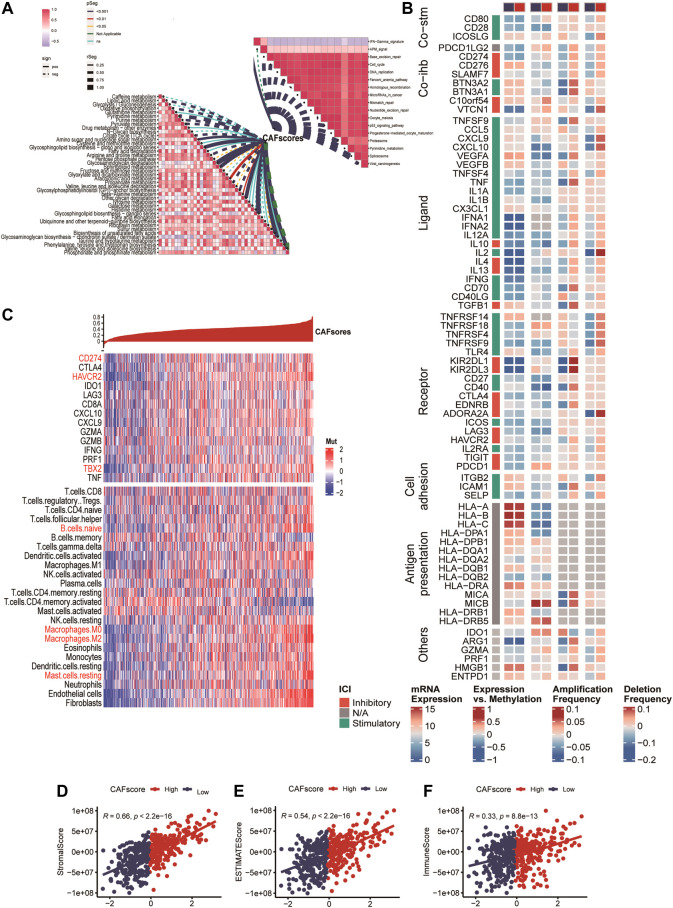
Immune analysis of the CAF-related scoring model. **(A)** Correlations between CAF score feature scores and metabolic pathways, immune-related pathways based on GSVA of GO and KEGG terms. **(B)** Multi-omics analysis of 75 immunomodulators between high and low CAF score samples. **(C)** Expression and Pearson correlations of immunity, ESTIMATE, stroma score, tumor purity, TIC, checkpoints and immune competence for each sample are illustrated in a heatmap.

We analyzed the correlation between the hypoCAFs signature score and several pathways, the score was negatively correlated with most of the metabolic-related pathways. It was also negatively correlated with some cancer-related pathways such as cell cycle, gene duplication, homologous recombination, and p53 signaling (Figure 6A).

After deconvolution analysis, samples with low CAF scores exhibited high levels of activated memory CD4 T cells and memory B cells, while samples with high CAF scores showed high expression levels of regulatory T cells and resting memory CD4 T cells. To evaluate immune competence, we examined the expression of immune checkpoints (CD274, CTLA4, HAVCR2, IDO1, LAG3) and immune competence factors (CD8A, CXCL10, CXCL9, GZMA, GZMB, IFNG, PRF1, TBX2, and TNF) (Figure 6B). The CAF score demonstrated a negative correlation with 14 out of 75 immunomodulators and 24 immune cells (Figure 6B). Finally, we compared the immune score (Figure 6D), ESTIMATE (Figure 6E), and stroma score (Figure 6F) between samples with high and low CAF scores, and observed that high CAF score samples exhibited elevated matrix and ESTIMATE scores.

### Immunotherapy prediction of metabolism-CAF score

To explore the role of CAF score in immunotherapy, we investigated the correlation between risk score and TMB. Our findings revealed that TMB expression was significantly higher in the low-risk subgroup than in the high-risk subgroup (Supplementary Figure S4). Moreover, to gain further insights into the nature of immunity in different risk subgroups, we analyzed genetic mutations. The top 20 genes with the highest mutation rates were identified in both the high-risk and low-risk subgroups (Supplementary Figure S4).

**FIGURE 7:**
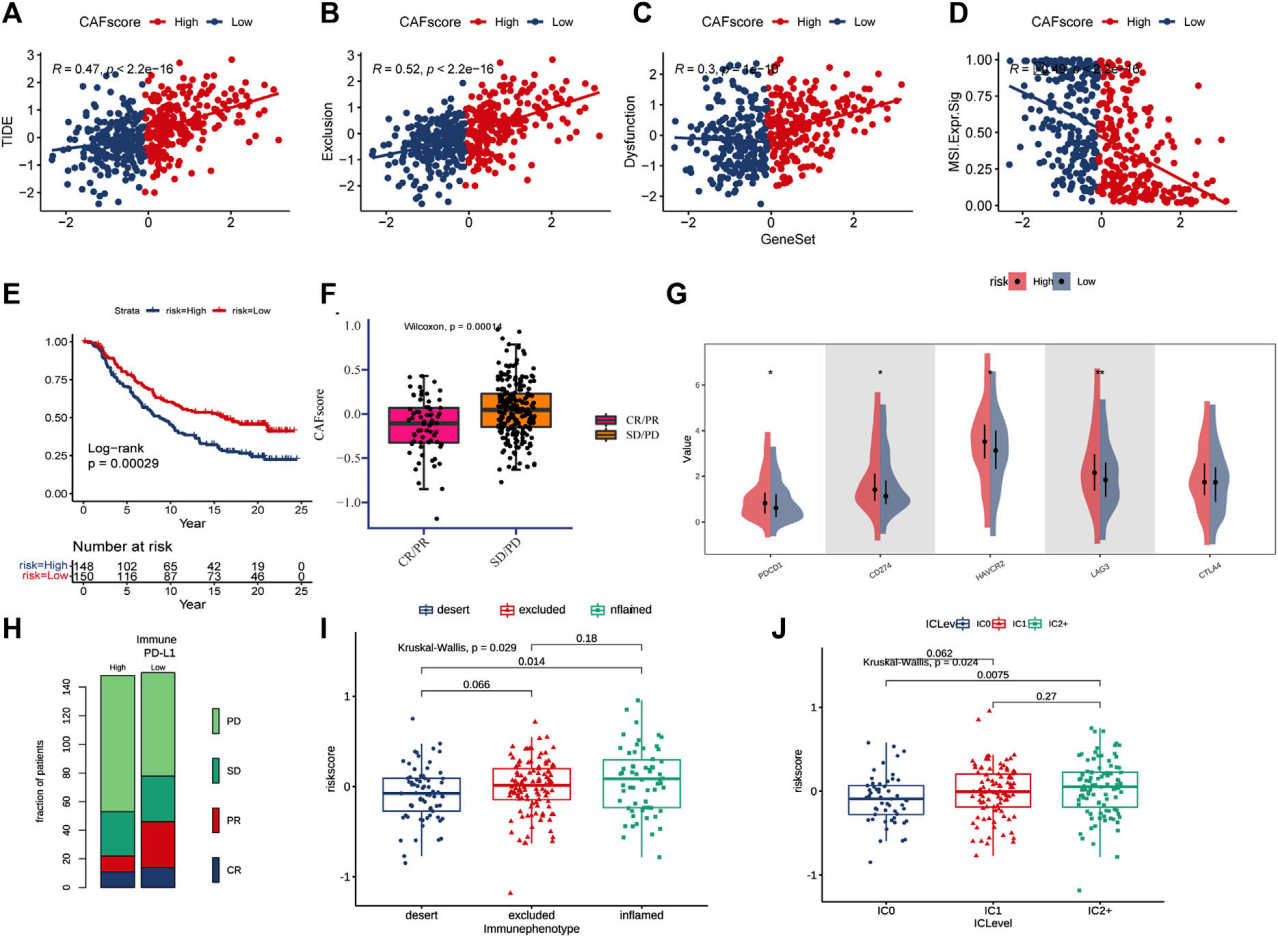
CAF score in predicting immunotherapy effect. **(A–D)** Correlations between CAF score and TIDE, dysfunction, exclusion, and MSI expression signatures. **(E)** Kaplan-Meier curve *versus* IMvigor210 survival analysis. **(F)** Wilcoxon test of anti-PD-L1 reactive CAF score variation. **(G)** Expression of 5 immune checkpoint molecules (PDCD1, CD274, CTLA4, LAG3, and HA VCR2) in high and low CAF score groups **(H)** Stacked histogram showing anti-PD-L1 between high and low CAF score difference in reactivity. **(I)** CAF score was tested at three scorch levels using the Kruskal–Wallis test. **(J)** Kruskal–Wallis test of the CAF score of PD-L1 expression on immune cells.

For ICB response prediction, we determined the correlations of CAF scores with TIDE, dysfunction, exclusion, and MSI Expr signature (Figures 7A–D). The results showed that CAF scores were positively associated with TIDE, dysfunction and exclusion, and negatively associated with MSI Expr sig. It was found that CRC patients with lower hypoCAFs scores had a higher possibility of responding to immunotherapy and may had better prognosis after immunotherapy, indicating that patients with lower CAFs scores were more likely to benefit from immune checkpoint therapy (Wilcoxon test, *p* = 0.0001, Figures 7E–H).

The expressions of five immune checkpoint molecules (PD1, PD-L1, CTLA4, LAG3 and HAVCR2) were compared between groups with high and low CAF score, the results showed that four immune checkpoint molecules (PD1, PD-L1, LAG3, and HAVCR2) were significantly upregulated in the high-risk group (Figure 7G). Furthermore, CAF score was strongly associated with the desert and inflamed immunophenotype (Kruskal–Wallis, *p* = 0.0029, Figure 7I). Our study also found that CAF score was positively correlated with PD-L1 expression in tumor cells and PD-1 expression in immune cells (Figure 7J).

### GDSC investigation of metabolism-CAF score

**FIGURE 8:**
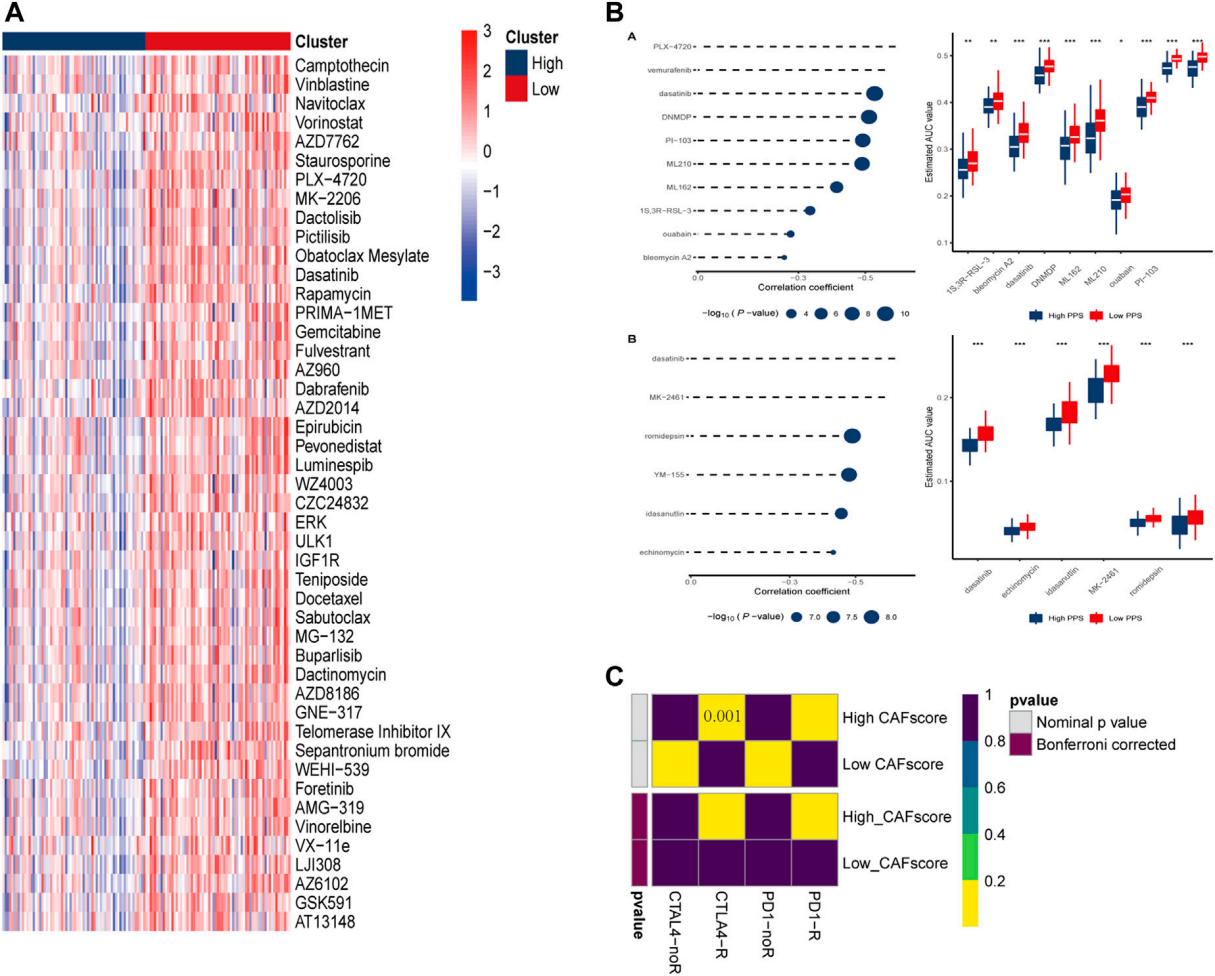
Prediction of drug and immune responses. **(A)** Heatmap showing IC_50_ estimates for high and low CAF scores. **(B)** Predicted and estimated AUC values for small molecule drugs in CTRP 2.0 and PRISM databases. **(C)** Immune responses to PD1 and CTLA4 in patients with high and low CAF scores.

Chemotherapy is the main treatment options for colorectal cancer liver metastases, therefor, whether CAF scores can accurately predict chemotherapy outcomes in colorectal cancer patients was investigated. GDSC is used to predict response to conventional chemotherapy in patients with colorectal cancer liver metastasis. A ridge regression model was built to predict IC_50_ of different drugs. We found that the IC_50_ of cisplatin, gemcitabine and other chemotherapeutics in the high CAF score group were significantly lower than those in the low CAF score group, suggesting that CAF score was positively correlated with chemotherapeutic drug sensitivity of colon cancer liver metastasis. In addition, we used the database to predict small molecule drugs (Figure 8B). The drugs vemurafenib, PLX-4720, dasatinib, and PI-103 were found to be negatively associated with the CAF score, with lower estimated AUC values in samples with high CAF score. These findings suggest that the predicted small-molecule drugs may be more effective in patients with high CAF score.

## Discussion

Growing evidence suggests that CAFs are key players in CRC metastasis. Meanwhile, metabolic reprogramming has profound effects on CAFs, thereby regulating cancer progression and metastasis, including glucose, glutamine and fatty acid metabolism (Zhu et al., 2022). A previous scRNA-seq study showed that CAFs identified in PDAC patients have a highly activated metabolic state. The new CAF subtype, called metabolic CAF (meCAF), uses glycolysis as the primary metabolic mode. Although PDAC patients with abundant meCAF have a higher risk of metastases, they have better immunotherapy responses when treated with programmed cell death protein 1 (PD-1) blockade (Wang et al., 2021). Downregulation of metabolism genes in CAFs of PDAC liver metastasis, but not those in lung metastasis, appeared to be regulated by DNA methyltransferase, CAFs metabolism modification may promote PDAC with organ-specific metastatic (Pan et al., 2021). However, the combined effects of fibroblasts with different metabolic status in colorectal cancer liver metastasis are unclear. Studying the role of metabotropic CAF-related gene signatures in the occurrence and development of colorectal cancer liver metastases may contribute to decode the mechanisms of liver metastases and guide appropriate treatment strategies for patients. Cancer-associated fibroblasts are important members of the TME, and previous studies have shown that CAFs with different molecular characteristics were classified into myCAF, pan-dCAF, pan-iCAF, pan-nCAF and pan-pCAF (Galbo et al., 2021). In our study, we identified prognostic CAF populations through single-cell transcriptomes, and these subtypes exhibited distinct activation of metabolism-related pathways, such as glucose metabolism, gluconeogenesis, cysteine and methionine metabolism, etc. In addition, we found that different CAF cells also exhibit extensive interactions with T cells, NK cells, and tumor cells through growth factors and cytokines, thereby promoting tumor progression (Jiang et al., 2021). Cellular communication has shown that the CXCL12-CXCR4/CXCR7 chemokine axis is expressed in hyperCAFs and is significantly expressed in metastases. CXCL12 not only binds to CXCR4, but also to CXCR3 and DPP4 on tumor cells in our study, which is consistent with previous reports (Jiang et al., 2021). Li et al. (Li et al., 2022) pointed out that inflammatory CAFs secrete IL6 and CXCL12 to chemoattract and regulate the function of T cells, which is similar to inflammatory CAFs found in other solid tumors. Costa et al. (Costa et al., 2018) identified differentially expressed secretory molecules, such as CCL11, CXCL12, CXCL13, and CXCL14, in CAF-S1 and CAF-S4 cells in breast cancer. CXCL12 can be produced by hyperCAF, the binding of CXCL12 to tumor cells can inhibit tumor cell apoptosis and change the characteristics of tumor cell adhesion (Augsten et al., 2014; Zhao et al., 2017). HyperCAFs also express the CXCL14, elevated CXCL14 levels in CAFs of clinical specimens are also associated with higher risks for disease recurrence and worse overall survival time in colorectal cancer (Zeng et al., 2013).

Many studies have reported the role of RUNX3 in inhibiting cancer cell migration and tumor growth (Kim et al., 2020). A study reveals that CAF-derived exosomal miR-17-5p promotes an aggressive phenotype in colorectal cancer by initiating a RUNX3/MYC/TGF-β1 positive feedback loop (Zhang et al., 2020). In another study, circMEttL3, which is transcriptionally activated by RUNX3, suppressed CRC development and metastasis by acting as a miR-107 sponge to regulate PER3 signaling (Zhang et al., 2022). Furthermore, RUNX3 has been shown to promote TRAIL-induced CRC apoptosis (Kim et al., 2019). CREB3L1 is a hypoxia-inducible cytokine (Mellor et al., 2013). Studies have shown that α-SMA-positive CAFs were activated through CREB3L1-mediated IL-1α production, the presence of CAF inhibits thyroid cancer growth and metastasis after CREB3L1 knockdown (Pan et al., 2022).

Cellular communication analysis and hub genes in CAF-related modules suggest the importance of MAST cells in the immune microenvironment remodeled by CAF (Johansson et al., 2010; Derakhshani et al., 2019). Mast cells can generate trypsin, TNF, IL-1, IL-6, and other factors to boost anti-tumor inflammatory responses, stimulate tumor cell apoptosis, and suppress cancer cell invasion (Ribatti and Crivellato, 2011). In prostate cancer, estrogen-induced CAF-derived CXCL12 binds to CXCR4 and enhances mast cell proliferation, migration, and inflammatory cytokine secretion, thus exhibiting oncogenic effects (Ellem et al., 2014). CAF plays a role in immunosuppression through various mechanisms, including collaborating with mast cells to promote the early malignant transformation of benign epithelial cells, and blocking DC maturation and antigen presentation (Cheng et al., 2016; Pereira et al., 2019).

To identify prognostic genes associated with CAFs and improve the clinical utility of the model, we utilized LASSO Cox regression and multivariate Cox regression analyses to identify key CAF-related genes with independent prognostic significance. The resulting risk score prognostic model for sexual symptoms was constructed based on 13 CAF-related genes that exhibited independent prognosis. Then, the model is validated in different GEO cohorts. ROC curve suggested that risk scores derived from genetic signatures were more effective in predicting overall survival at 1-, 3-, and 5-year survival. After performing functional analysis, we observed that several cancer-related pathways and activated cellular crosstalk pathways were enriched in samples with high CAF score. We also investigated the expression levels of immunosuppressive gene markers and found an association between CAF score and immune checkpoint molecules (CXCL9, CTLA4, CD274, TNF, TBX2). Finally, we analyzed chemotherapeutic drug resistance and sensitivity data to predict potential correlation of CAF score and therapeutic effects of chemotherapeutic drugs. These results suggest that the CAF signature is a potential clinical model for the determination of whether a CRC patient are more likely to respond to ICIs or chemotherapeutics.

Moreover, our study identified CD274, HAVCR2, and TBX2 as potential targets for immunotherapy of CRC liver metastasis, as they were found to be increased in samples with high CAF scores. Previous study has reported blocking these immune checkpoints may represent a promising strategy for HCC treatment (Lian et al., 2020). Our results suggest that patients with high CAF scores may have higher sensitivity to some small-molecule drugs. For instance, in a recent clinical trial, the addition of vemurafenib improved the progression-free survival in patients with BRAF-mutant metastatic colorectal cancer treated with irinotecan and cetuximab (Kopetz et al., 2021). While our model showed good predictive performance for the sensitivity of colorectal cancer to cisplatin, it may not be as effective in predicting sensitivity to classical chemotherapeutic agents like oxaliplatin, fluorouracil, and irinotecan. Nonetheless, our findings can guide the selection of chemotherapy drugs for some colorectal cancer patients. In summary, our risk score model provides better prediction of the sensitivity of colorectal cancer patients to immunotherapy and has potential as a reference for selecting appropriate immunotherapy regimens for these patients.

However, there are limitations to our study. First, the data used in this study were obtained from online databases like TCGA and GEO, and further validation study with a larger sample size is required. Second, the findings of this study need to be prospectively validated in a cohort of colorectal cancer liver metastasis patients who receive immunotherapy.

In conclusion, our study indicates that metabolically active CAFs have a stronger communication and interaction with immune and tumor cells compared to metabolically suppressed CAFs. Moreover, hypoCAF score has better prognostic efficacy than hyperCAF score in terms of overall survival and recurrence-free survival of patients with metastatic colorectal cancer. We have developed a metabolic-CAF score model based on hypoCAF score and verified its ability to predict the prognosis of patients with metastatic colorectal cancer. The CAF score can predict the sensitivity of colorectal cancer liver metastasis chemotherapy drugs and is correlated to the prediction of immunotherapy outcomes. Our study provides new ideas and research methods for understanding the metabolic characteristics of CAFs and their role in patients with metastatic colorectal cancer. This study improves the treatment of colorectal cancer metastasis, and further exploration of the mechanism of CAFs can provide a theoretical basis and potential drug targets for CRC patients with metastasis.

## Data Availability

Publicly available datasets were analyzed in this study. This data can be found at the Gene Expression Omnibus (GEO) database (https://www.ncbi.nlm.nih.gov/geo/) - Accession number: GSE178318.
