# Supplementary material for: Metabolic-suppressed cancer-associated fibroblasts limit the immune environment and survival in colorectal cancer with liver metastasis
Source: Front Pharmacol. 2023 Aug 31;14:1212420. doi: 10.3389/fphar.2023.1212420 (PMC10501863; doi:10.3389/fphar.2023.1212420)

Supplementary Fig1: (A) Proportion of hypoCAFs and hyperCAFs in different sexes. (B) Proportion of hypoCAFs and hyperCAFs in metastases and primary sites. (C) Proportion of hypoCAFs and hyperCAFs in chemotherapy and non-chemotherapy. (D) COL07 , COL12 , COL15, COL16, COL17 and COL18 the ratio of hypoCAFs and hyperCAFs per patient. (E) UMAP map of metastases and primary sites. (F) UMAP map of distribution of 6 samples.


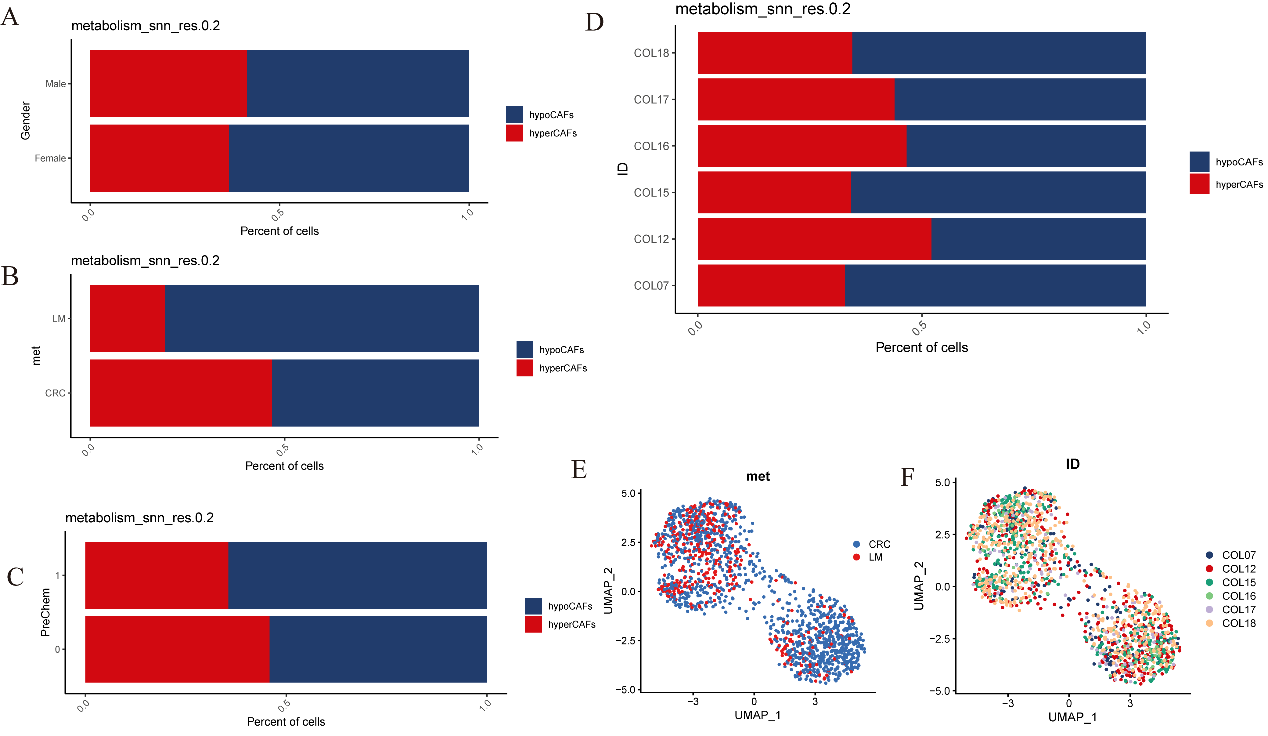


Supplementary Fig2: (A) umap map of distribution of CRABP2, PLA2G2A, OGN and MGST1 metabolic genes in hypoCAFs and hyperCAFs.(B) Comparison of four genes in hypoCAFs and hyperCAFs.


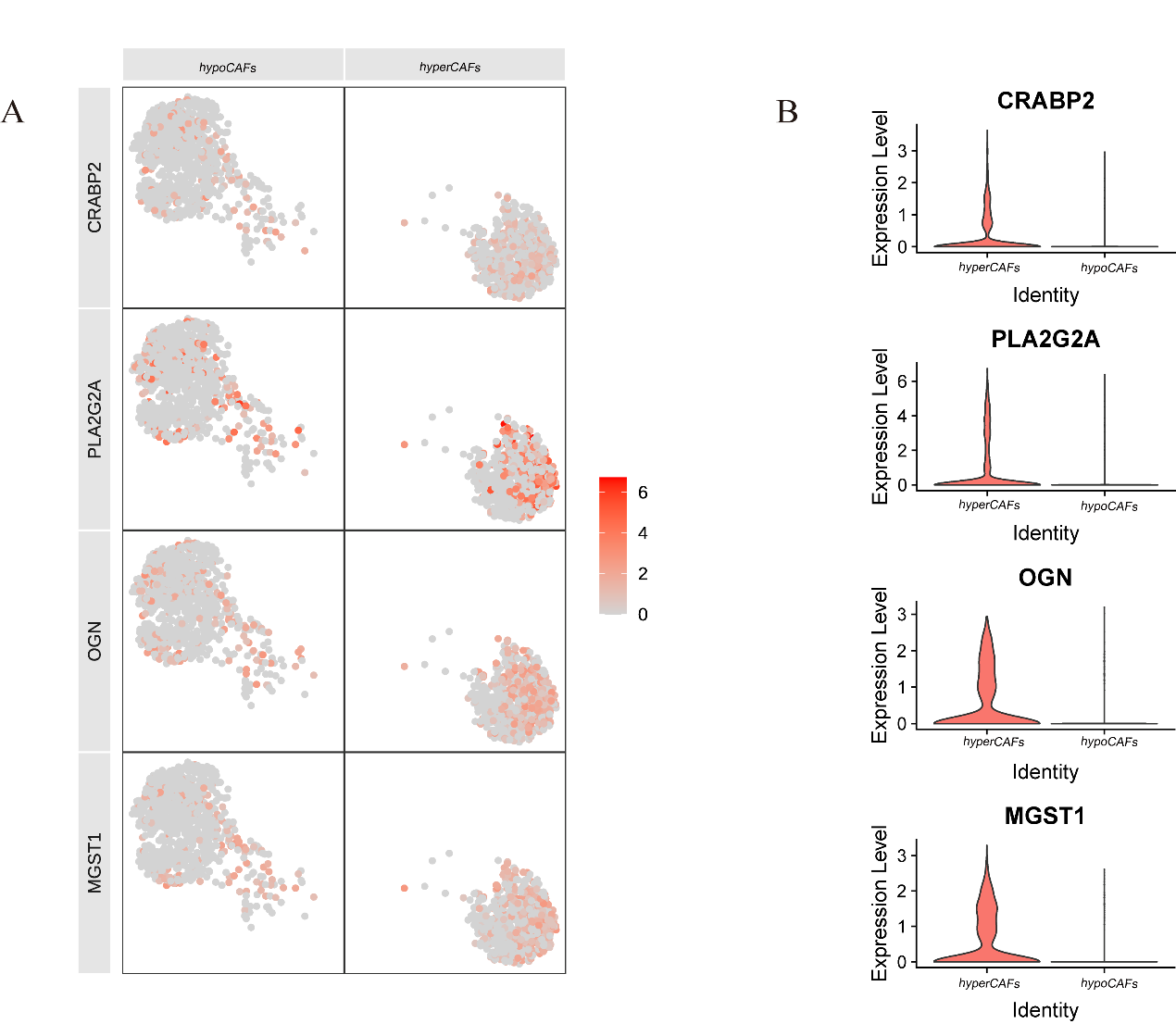


Supplementary Fig3 (A) Boxplot showing the difference between primary and metastatic lesions in hypoCAF and hyperCAF. (B)The activation track of CAF is divided into two states (CAF State0/1). (C) Cellular communication of hypoCAFs and hyperCAFs under CXCL signaling.


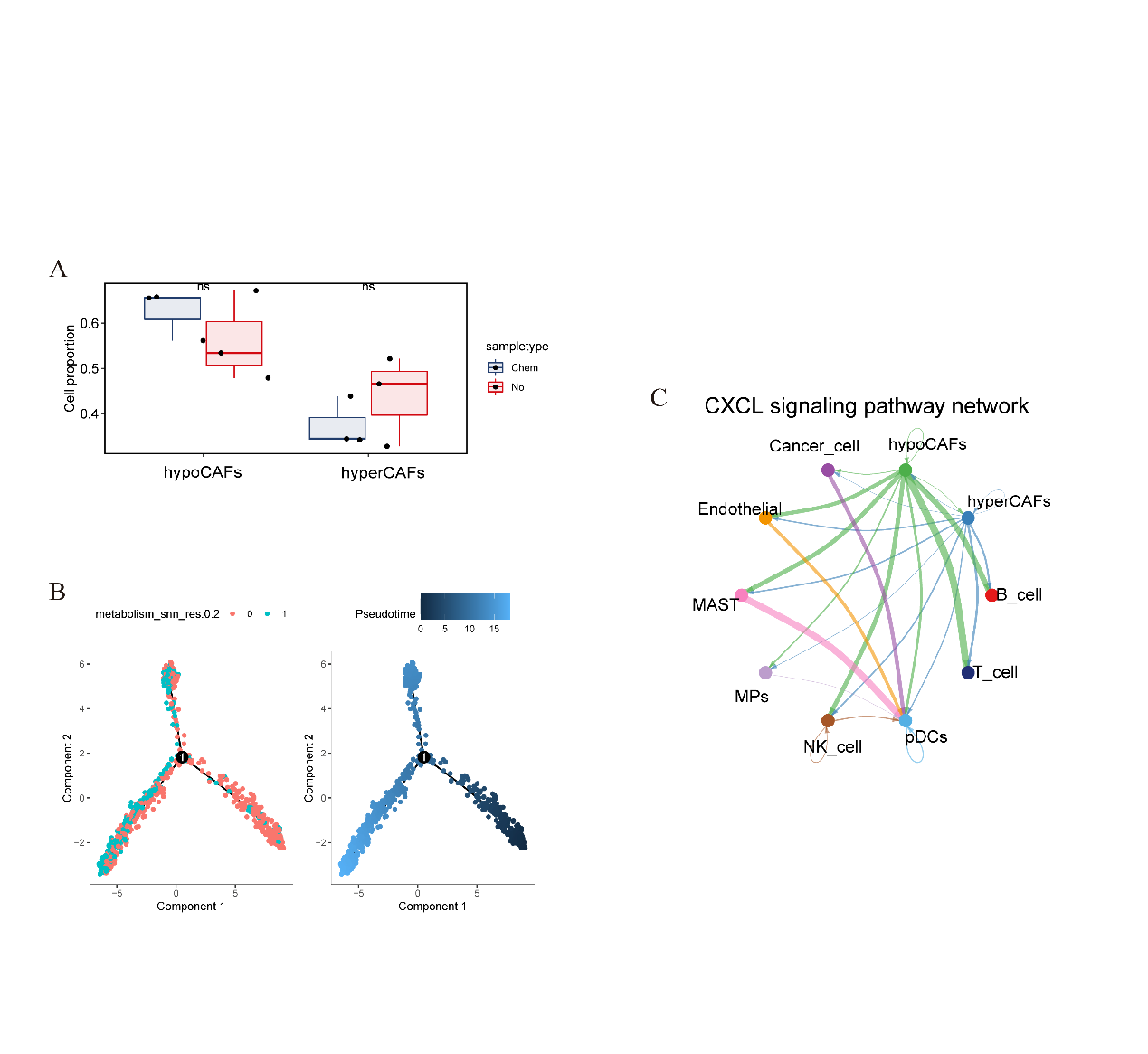


Supplementary Fig4 Waterfall plot of the mutation distribution of the top 20 most frequently mutated genes in CRC patients in the TCGA dataset. Comparison of top 10 mutated genes between low group and high group.
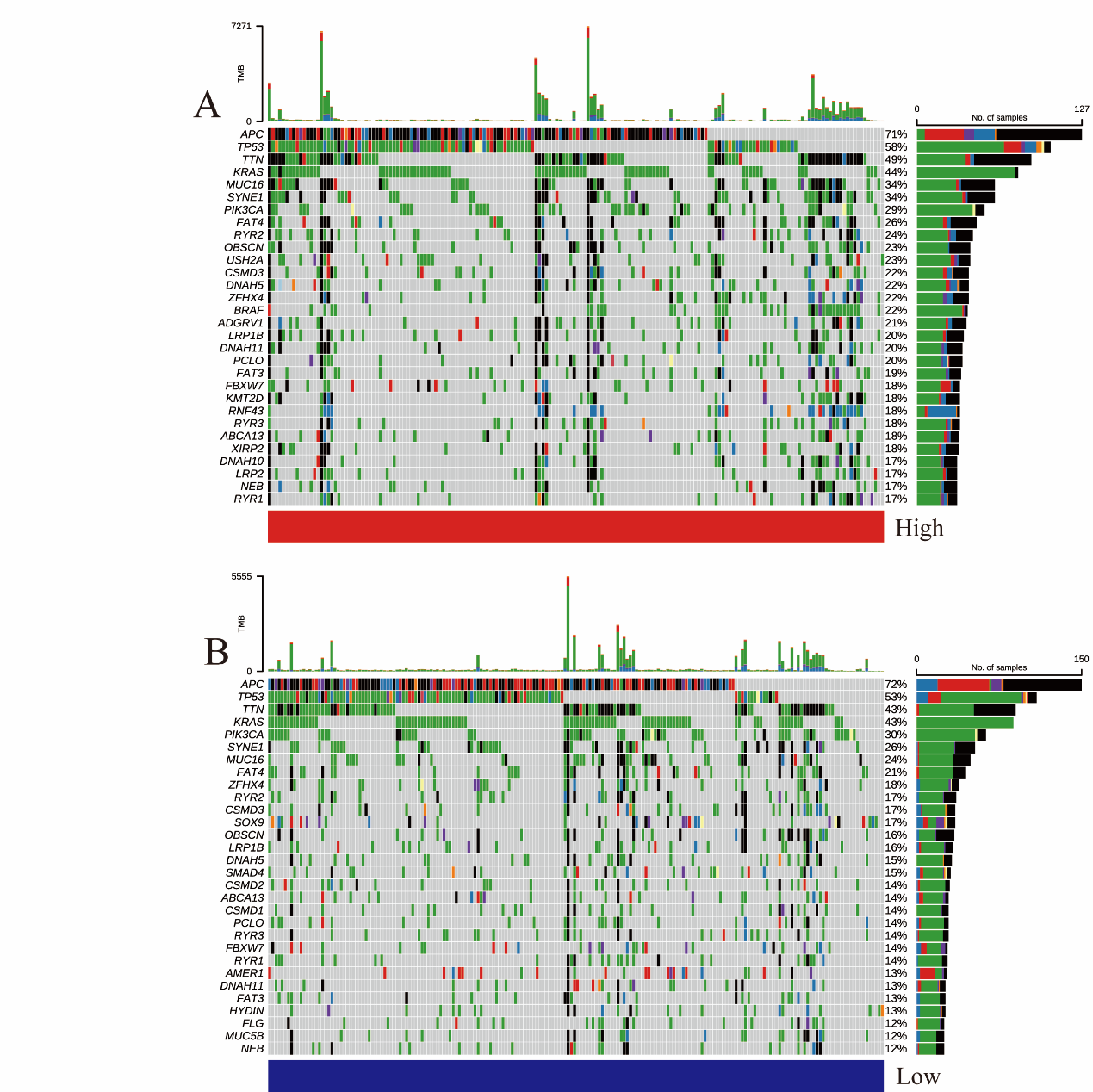

Supplement: Supplementary file 2 [file DataSheet2.docx]
